# The Osteopathic Bill

**Published:** 1902-03

**Authors:** 


### The Osteopathic Bill.

THE perennial effort before the legislature to impair, in some manner, the efficiency of the medical practice act in the State of New York has failed at least in one particular this year. The much boasted effort of the osteopaths to become doctors without the training and education required of doctors has come to naught.

The bill introduced by Senator Brackett early in the session, but for which he disclaimed responsibility, was brought to a hearing before the Senate Judiciary Committee January 29, 1902. The committee was addressed in opposition by Bishop Doane, Dr. A. Jacobi, Dr. A. Vander Veer, Dr. Henry R. Hopkins, Dr. E. Eliot Harris and some others. Dr. Frank Van Fleet, chairman of the legislative committee of the State Medical Society conducted the hearing on the part of the opposition. The proponents of the measure were represented principally by osteopathist Steele and former Governor Fisk, of Vermont.

The senate chamber, where the hearing was held, presented an interesting and animated scene, filled as it was on floor and in galleries. It was not difficult for the opponents of the bill to convince the committee that it would be unwise to pass it. The arguments were so conclusive that without leaving their seats the committee by a vote of 7 to 2 decided to bury the bill. The dignified manner in which the bill was opposed and the good temper of the speakers was the subject of comment on all sides —even the osteopathic sympathisers conceding this much—all of which could not fail to make a favorable impression. It is to be hoped that a similar spirit will prevail in all legislative hearings whenever questions of medical interest are involved.

